# Listening effort and fatigue among cochlear implant users: a scoping review

**DOI:** 10.3389/fneur.2023.1278508

**Published:** 2023-11-03

**Authors:** Cato Philips, Laure Jacquemin, Marc J. W. Lammers, Griet Mertens, Annick Gilles, Olivier M. Vanderveken, Vincent Van Rompaey

**Affiliations:** ^1^Experimental Laboratory of Translational Neurosciences and Dento-Otolaryngology, Faculty of Medicine and Health Sciences, University of Antwerp, Antwerp, Belgium; ^2^Department of Otorhinolaryngology/Head and Neck Surgery, Antwerp University Hospital, Antwerp, Belgium; ^3^Department of Education, Health and Social Work, University College Ghent, Ghent, Belgium

**Keywords:** cochlear implantation, listening effort, fatigue, listening-related fatigue, scoping review, bilateral hearing loss

## Abstract

**Introduction:**

In challenging listening situations, speech perception with a cochlear implant (CI) remains demanding and requires high levels of listening effort, which can lead to increased levels of listening-related fatigue. The body of literature on these topics increases as the number of CI users rises. This scoping review aims to provide an overview of the existing literature on listening effort, fatigue, and listening-related fatigue among CI users and the measurement techniques to evaluate them.

**Methods:**

The Preferred Reporting Items for Systematic Reviews and Meta-Analyses (PRISMA) Statements were used to conduct the scoping review. The search was performed on PubMed, Scopus, and Web of Science to identify all relevant studies.

**Results:**

In total, 24 studies were included and suggests that CI users experience higher levels of listening effort when compared to normal hearing controls using scales, questionnaires and electroencephalogram measurements. However, executing dual-task paradigms did not reveal any difference in listening effort between both groups. Uncertainty exists regarding the difference in listening effort between unilateral, bilateral, and bimodal CI users with bilateral hearing loss due to ambiguous results. Only five studies were eligible for the research on fatigue and listening-related fatigue. Additionally, studies using objective measurement methods were lacking.

**Discussion:**

This scoping review highlights the necessity for additional research on these topics. Moreover, there is a need for guidelines on how listening effort, fatigue, and listening-related fatigue should be measured to allow for study results that are comparable and support optimal rehabilitation strategies.

## 1. Introduction

Hearing loss is a sensory deficit that has already been investigated extensively due to the well-known communication difficulties experienced by the hearing-impaired population in their daily life. When hearing thresholds deteriorate, communication difficulties and the impact on daily life may increase with, presumably, the highest impact for patients with severe-to-profound hearing loss. Cochlear implantation (CI) has become the standard treatment for patients with this degree of hearing loss (1).

The outcome of CI is frequently investigated using Patient-Reported Outcome Measures (PROMs) and speech comprehension tests (2). Research on health-related quality of life (HRQoL) using PROMs such as the Nijmegen Cochlear Implant Questionnaire and the Abbreviated Profile of Hearing Aid Benefits have observed significant improvement in levels of HRQoL after CI (3, 4). The speech recognition abilities can be evaluated using speech comprehension tests with words or sentences either in quiet or in noise. To date, improvements in speech perception after CI are already demonstrated (5).

Although satisfactory speech comprehension in quiet can be reached with a CI, regardless of the large inter-individual differences between CI users, the electric way of listening remains different from acoustic listening. An adequate frequency resolution is necessary in various listening situations but can only be provided to a limited extent by the CI, leading to difficulties in understanding speech in noise (6). Such adverse listening situations may lead to increased levels of listening effort and subsequent listening-related fatigue among CI users.

Listening effort is defined by Pichora-Fuller et al. (7) as “the deliberate allocation of mental resources to overcome obstacles in goal pursuit when carrying out a task, with listening effort applying more specifically when tasks involve listening”. Both subjective and objective (i.e., physiological and behavioral) measurements can be used to assess listening effort. Previous research used visual analog scales (VAS), Likert scales, or questionnaires such as the Speech, Spatial, and Qualities of Hearing scale (SSQ) to investigate the subjectively perceived listening effort, as these subjective measurements are quick and easy to complete and administer (8, 9). Both behavioral measurements, e.g., reaction time and dual-task paradigms, and physiological methods, e.g., pupillometry, electroencephalography (EEG), event-related potentials (ERP), and functional magnetic resonance imaging, objectively evaluate listening effort (10–14).

General fatigue (further referred to as fatigue) is defined by Hornsby et al. (15) as “a general feeling of being tired, worn out, or sluggish” although a number of definitions are used in the literature. Listening-related fatigue on the other hand is the result of prolonged and high levels of listening effort (16). Both terms are used interchangeably in the literature and have been found to be important contributors to reduced QoL, cognitive abilities, and workplace productivity (15, 17). Fatigue and listening-related fatigue can be investigated using subjective methods such as focus group discussions and questionnaires, e.g., Profile of Mood States (POMS), the Fatigue Assessment Scale (FAS), and the Vanderbilt Fatigue Scale for Adults (VFS-A), and objective methods including evaluation of task performance, biological markers, and (electro)physiological measurements (18–21).

The large inter-individual differences in speech comprehension and the finding that only 22% of the variance can be explained by pre-, per-, and postoperative factors (e.g., pure tone average, duration of hearing loss, surgical approach and brand) lead to an increasing interest into the influencing factors of the CI outcome to optimize rehabilitation (22). No research has yet examined the role of listening effort on inter-individual differences.

To provide proper rehabilitation, it is important to further investigate listening effort, fatigue, and listening-related fatigue among CI users and the different measurement methods available to examine this. To date, two reviews have been published (23, 24). Ohlenforst et al. (24) conducted a systematic review of listening effort and included two studies that investigated this among CI users. The review by Holman et al. (23) could only include three studies that examined fatigue among CI users. The two studies concluded that the small number of included studies and their heterogeneity resulted in inconclusive findings. Moreover, the reviews did not primarily focus on cochlear implants. Given the increasing body of literature and growing number of studies on these topics, it is crucial to provide an update. Therefore, the aim of this scoping review is to provide an overview of the current knowledge regarding listening effort, fatigue, and listening-related fatigue among adult CI users, as well as the various measurement methods to investigate this.

## 2. Materials and methods

**Figure 1:**
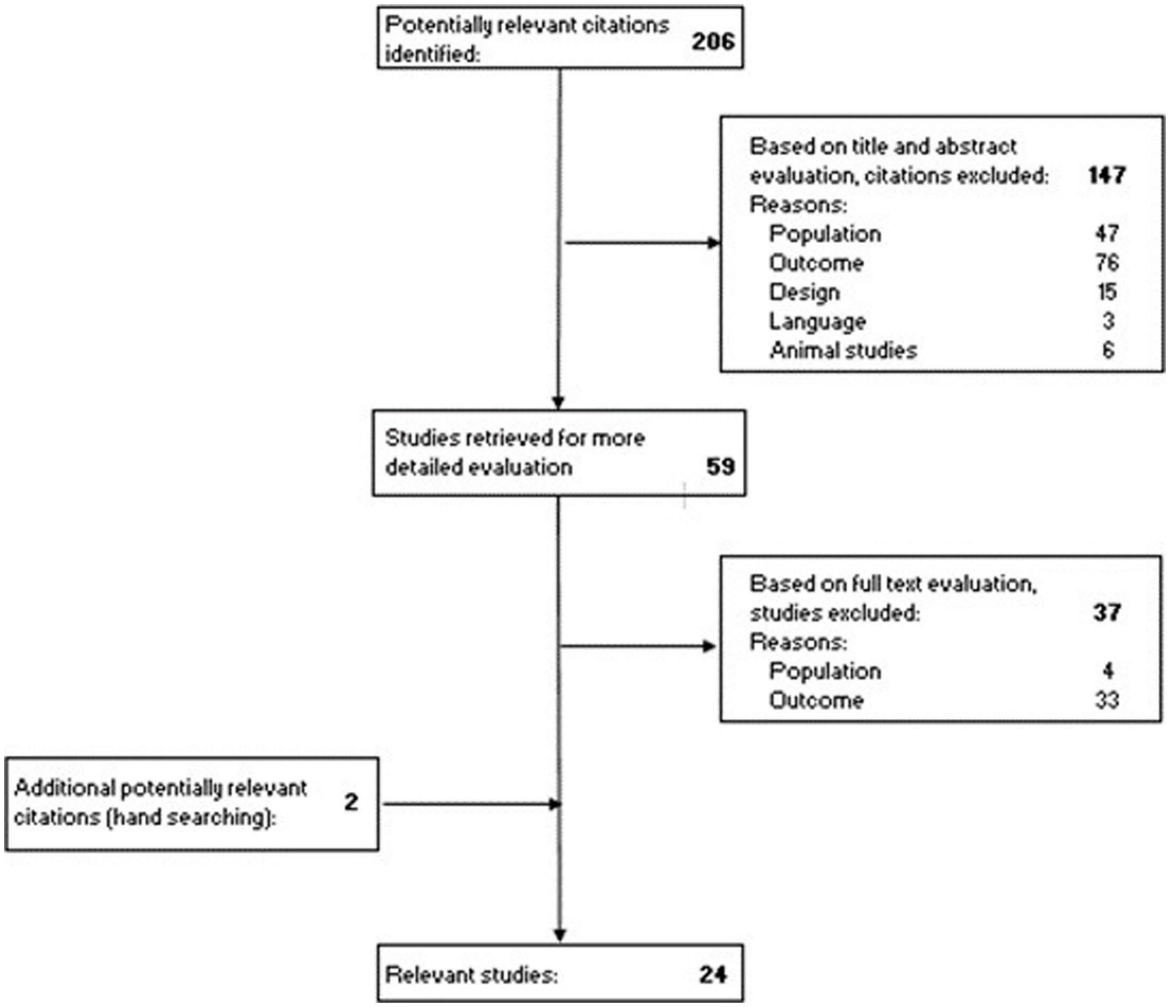
PRISMA flow diagram of the search strategy.

The scoping review was preregistered on 7 June 2022 at the Open Science Framework (OSF) Registries (10.17605/OSF.IO/QP68U). The Preferred Reporting Items for Systematic Reviews and Meta-Analyses (PRISMA) Statements and the framework of Arksey and O’Malley were used to identify all relevant research studies (25, 26). An extensive search was carried out on 11 May 2022 and 10 January 2023 using the following databases: PubMed, Scopus, and Web of Science. Studies that investigated listening effort, fatigue, and/or listening-related fatigue among adults with CI were included. No distinction was made to exclude studies based on whether the CI users had unilateral or bilateral, prelingual or acquired hearing loss, or were unilaterally or bilaterally implanted. An overview of the search strategy for the PubMed database is represented in Table 1. After removing all duplicates, 206 abstracts were screened. Studies were excluded if the studies included a different study population (e.g., people with normal hearing, children, animals, participants without CI), did not investigate listening effort, fatigue, or listening-related fatigue (e.g., quality of life and speech intelligibility), or were protocol or congress papers. The influencing factors on listening effort, fatigue, and listening-related fatigue such as patient-related variables and device-related factors related to CI hardware and software are beyond the scope of this review. As a result, studies that investigated solely the influencing factors were excluded as well. Studies in languages other than English or Dutch were excluded. After the first screening stage, full texts were read resulting in 22 included studies. Hand-searching revealed two additional studies, bringing the number of included studies to 24. The search strategy is presented in detail in Figure 1.

**Table 1:** Overview of the search strategy PubMed database.

“Cochlear Implants”[Mesh] OR “Cochlear implantation”[Mesh] OR “Cochlear implant”[tiab] OR “Cochlear implant*”[tiab] OR “Cochlear implantation”[tiab]	AND	“listening effort”[Mesh] OR “listening effort”[tiab] OR “listening-related fatigue”[tiab] OR “fatigue”[Mesh] OR “fatigue”[tiab] OR “mental fatigue”[Mesh] OR “mental fatigue”[tiab] OR “auditory fatigue”[Mesh] OR “auditory fatigue”[tiab]

## 3. Results

Out of the 24 included studies, 3 studies had a qualitative study design and 21 studies had a quantitative study design. A total of 19 studies evaluated listening effort and 3 studies investigated fatigue and/or listening-related fatigue. Two studies included both listening effort and fatigue or listening-related fatigue in their study. All studies are presented in Appendix 1 where author, year of publication, number of participants, measurement methods, and outcome are described. These studies will be discussed in detail in this section, starting with the studies that examined listening effort followed by the studies that investigated fatigue and listening-related fatigue.

### 3.1. Listening effort

Twenty-one studies documented listening effort, the increased effort that is needed when listening, among CI users. Both subjective and objective measurements were used in the included studies to investigate this. Subjective measurements including focus group discussions, scales, and questionnaires were used in 18 studies. In nine studies, listening effort was objectively evaluated with dual-task paradigms, EEG, and pupillometry. Table 2 provides an overview of the different measurement techniques used in the included studies.

**Table 2:** Information on the subjective and objective listening effort measurement methods used in the included articles.

Subjective measurement methods	Focus group discussion	An interactive discussion led by a moderator where participants with the same background or shared experience discuss a specific topic (27).
	Visual analog scale (VAS)	A measurement instrument to investigate clinical phenomena as a range across a continuum (28).
	Speech, Spatial, and Qualities of Hearing scale (SSQ)	Questionnaire consisting of three scales (speech comprehension, spatial hearing, and quality of sound scale) with a VAS as a response option. Listening effort is questioned as a subscale of the qualities of the sound scale (29).
	Effort Assessment scale	VAS comprised of three questions of the SSQ and three additional questions for the evaluation of listening effort (18).
	Likert scale	A scale that measures the agreement or disagreement of a statement (30).
	Adaptive Categorical Listening Effort Scaling (ECALES)	A 7-point Likert scale (31)
	Cochlear Implant-related Quality of Life (CIQOL) item bank	Questionnaire consisting of 81 5-point Likert scale items including a listening effort domain (32).
	NASA Task Load Index	A 10-point scale to answer the question: “How hard did you have to listen to accomplish your level of performance in that block?” (33).
Objective measurement methods	Dual-task paradigm	An objective behavioral measurement that requires the participant to perform two tasks, a primary listening task and a secondary visual reaction time task. Both tasks are performed separately and simultaneously in order to calculate listening effort (34).
	Pupillometry	An objective measurement method that measures changes in the task-evoked pupil dilation. An increased pupil dilation is seen when the effort needed increases (35).
	Electroencephalograms (EEG)	Direct measurement of the brain activity (36). With the use of scalp electrodes, the electrical brain activity will be measured (37).

Hughes et al. (38) organized a focus group discussion for the development of a theoretical framework of listening effort in adult CI users (*n* = 9), severe-to-profound hearing aids users (*n* = 4) waiting for their cochlear implantation, and spouses with normal hearing (*n* = 2). Hughes et al. (38) concluded that listening effort is a complex, multidimensional construct and defined it as “the mental work undertaken when: (1) attending to the auditory signal, (2) processing auditory information, and (3) adapting to and compensating for the hearing loss.”

#### 3.1.1. Listening effort in CI users compared with controls with normal hearing

Eight studies compared listening effort between CI users and controls with normal hearing.

##### 3.1.1.1. Scales and questionnaires

Based on subjective measurements, listening effort among CI users seems to be higher compared to controls with normal hearing (11, 18, 39–42). Abdel-Latif and Meister (10) could confirm a higher listening effort for CI users compared to participants with normal hearing when measuring listening effort at the same signal-to-noise ratio (SNR). When a speech intelligibility of 50% and 80% was targeted in CI users and controls with normal hearing, listening effort did not differ between both groups (10). When measuring at the same performance level, the SNR differed between both groups with higher SNRs for the CI group compared to controls with normal hearing, which may explain the lack of difference between both groups (10). Although different measurement methods were used in these studies, similar results were established. The measurement method of each study is presented in Appendix 1. The similar outcome in those studies may indicate that subjective measurements can be useful to investigate listening effort in this population. It might, therefore, also be useful to conduct these subjective measurements in addition to the traditional hearing assessment, provided that further research is conducted to determine the most appropriate scale or questionnaire to use.

##### 3.1.1.2. Electroencephalogram

Finke et al. (11) executed EEG to investigate the relationship between listening effort and endogenous ERP during the execution of an auditory oddball paradigm. According to the results based on the subjective measurement, Finke et al. (11) found a higher listening effort in CI users compared to controls with normal hearing as prolonged higher-order processing measured with the P3 was found in CI users.

##### 3.1.1.3. Pupillometry

Wagner et al. (14) concluded that pupil dilation differed in response magnitude, time course, morphology, latencies, and number of peak dilations among the included unilateral CI users. Compared to participants with normal hearing, the function of pupil dilation differed in time course, shape, and location and the phasic event-related pupil dilation was smaller and decreased at a slower rate in CI users, which might suggest a difference in exerted effort for speech processing between both groups (14).

##### 3.1.1.4. Dual-task paradigm

Abdel-Latif and Meister (10) and Perreau et al. (42) could not confirm differences in listening effort between CI users and controls with normal hearing executing dual-task paradigms. The primary task in the study of Abdel-Latif and Meister (10) was a speech recognition task at a performance level of 80% while for the secondary task, the participant had to push a button when a white cross appeared on the computer screen. Perreau et al. (42) executed the Hearing-in-Noise Test (HINT) consisting of 20 sentences at a performance level of 50% at 6 SNR conditions as the primary task and the Stroop test, a test where the participant had to respond in which color the written color is presented, as a secondary task (43). This contradictory finding with the other measurement methods might suggest that the executed dual-task paradigms, especially the secondary tasks, might not be sensitive enough to measure the subtle mechanisms of listening effort between both groups (10).

#### 3.1.2. Listening effort in CI users compared with hearing impaired controls and among different CI groups

##### 3.1.2.1. Scales and questionnaires

Dwyer et al. (40) and Alhanbali et al. (18) investigated the difference in listening effort between CI users and hearing aid users. They found no difference in subjective listening effort between CI users, hearing aid users, and single-sided deafness (SSD) based on the SSQ and the Effort Assessment scale (18, 40). These findings may suggest that self-reported listening effort cannot be predicted by the hearing level, that hearing level is not related to the self-reported listening effort, or that the questionnaires used are not sensitive enough to infer a difference between hearing-impaired groups (18).

Several studies investigated subjective listening effort among different CI groups with contradictory findings possibly caused by differences in study population and subjective measurement methods. No difference in listening effort was found between bilateral and bimodal CI users, between unilateral and bimodal CI users, and between unilateral, bilateral, and short-electrode CI users based on the Effort Assessment scale and the SSQ (8, 42, 44). Noble et al. (9), who also executed the SSQ, concluded that bilateral CI users might experience less listening effort compared to unilateral and bimodal CI users. This finding was confirmed by McRackan et al. (32) measuring listening effort with the Cochlear Implant-related Quality of Life (CIQOL) item bank. Furthermore, Gifford et al. (45) concluded that patients with bilateral electric-acoustic stimulation experience lower levels of listening effort compared to bimodal CI users based on their VAS ratings of listening difficulty. Devocht et al. (46) reported that the decline of listening effort with increasing SNR is bigger for bimodal CI users than for unilateral CI users. The results of Noble et al. (9), McRackan et al. (32), and Devocht et al. (46) are in consistency with the finding that better hearing preservation in the implanted ear will result in better speech understanding (45).

Studies investigating the impact after cochlear implantation all executed the full version of the SSQ (3, 9, 40, 47). After cochlear implantation, subjective listening effort seems to decrease in CI users with SSD and with asymmetric hearing loss (3, 47). Dwyer et al. (40) reported a significant decrease in listening effort in bilateral and bimodal CI users compared to SSD patients, while Noble et al. (9) stated that listening effort decreases after unilateral or bilateral implantation but would not decrease significantly in bimodal CI users. The lack of a significant decrease in bimodal CI users after implantation is somehow unexpected and therefore Noble et al. (9) stated that further research is necessary in the specific patient group of bimodal CI users.

##### 3.1.2.2. Dual-task paradigm

In accordance with their subjective measurement method, Sladen et al. (44) could not find a difference in listening effort between bilateral and bimodal CI users using a dual-task paradigm, and no significant difference in listening effort between unilateral and bimodal CI users was found by Yüksel et al. (48). However, Perreau et al. (42), who established a difference in listening effort between unilateral and bilateral CI users based on their subjective measurement, could not confirm this by executing their dual-task paradigm. The sensitivity of the secondary task might have influenced these results (10).

##### 3.1.2.3. Pupillometry

Burg et al. (49) compared listening effort between the bilateral and unilateral conditions in 12 CI users. An increased pupil dilation, indicating higher listening effort, was found for the bilateral condition compared to the unilateral condition (49). With increasing task performance, pupil dilation increases. The increased task engagement for the bilateral condition might explain the higher listening effort for this condition (49).

##### 3.1.2.4. Electroencephalogram

Dual-task paradigms and pupillometry are indirect measures of brain activity, whereas EEG measurements may lead to a better knowledge of the brain regions responsible for increased listening effort (36). Dimitrijevic et al. (36) investigated the relationship between listening effort, alpha power, and neural entrainment measuring EEG when executing the digits-in-noise (DIN) test, in which the participant had to repeat three spoken digits in the presence of background noise (50). The alpha power may be a neural correlate of listening effort and neural entrainment relates the acoustic feature fluctuations with the brain activity fluctuations (12, 36). Dimitrijevic et al. (36) concluded that higher left frontal inferior frontal gyrus (IFG) alpha power results in an increment of subjective listening effort measured with the NASA Task Load scale. Furthermore, the coherence of the speech envelope between the auditory cortex and the envelope of typical human syllables situated at the 2–5 Hertz (Hz) range was investigated. Dimitrijevic et al. (36) found that listening effort increases with a lower coherence between the speech envelope and the auditory cortex in the 2–5 Hz range. Paul et al. (12) investigated the relationship between subjective listening effort measured with a 10-point scale and the within-subject variability of cortical alpha power. In contrast to the study of Dimitrijevic et al. (36) and Paul et al. (12) found that the left frontal IFG alpha power declines when subjective listening effort increases although this result was not significant. This contradictory finding may be due to differences in study design and signal processing (12). Furthermore, Paul et al. (12) found that the alpha power in the parietal brain regions seems to be higher for medium subjective listening effort while a lower alpha power was found for lower and higher subjective listening effort (12).

Speech-sound processing in CI users can be measured using auditory ERP, more specifically with the N1–N2 complex. Finke et al. (11) executed an auditory ERP measurement to investigate the relationship between the neural processing of words in different background conditions, the verbal abilities of CI users, and speech intelligibility. They found a relationship between the ERP measurements and the subjective listening effort, with higher listening effort ratings being associated with prolonged N2/N4 latencies (11).

#### 3.1.3. Correlation between subjective and objective measurements

Two of the studies that used both subjective and objective measurements to investigate listening effort discussed the correlation between both measurements. Abdel-Latif and Meister (10) and Perreau et al. (42) found no correlation between their Adaptive Categorical Listening Effort Scaling (ECALES) and 10-point scale and their dual-task paradigm. The low sensitivity of the secondary task of the dual-task paradigm might be a reason why no correlation between the subjective and objective measurement could be found (10). On the other hand, this lack of correlation might suggest that different measurement methods tap into different underlying dimensions of listening effort (10, 51).

### 3.2. Fatigue and listening-related fatigue

Fatigue is “a general feeling of being tired, worn out, or sluggish” (15), while prolonged listening in situations that require a lot of listening effort may lead to *listening-related fatigue* (16). Five studies documented fatigue and/or listening-related fatigue among CI users. The different measurement techniques used in these studies are presented in Table 3.

**Table 3:** Measurement methods of fatigue and listening-related fatigue used in the included articles.

Subjective measurement methods	Focus group discussion	An interactive discussion led by a moderator where participants with the same background or shared experience discuss a specific topic (27).
	Fatigue subscale of the Profile of Mood States (POMS)	A validated questionnaire with 65 statements and 5-point Likert scales as response options. The fatigue mood state is one of the six dimensions of mood swings (52).
	Fatigue assessment scale	A 10-item scale with a five-point Likert scale as a response option, resulting in a score between 0 and 40. Higher levels of fatigue are presented with a higher score on the FAS (21).
	Likert scale	A scale that measures the agreement or disagreement of a statement (30).

#### 3.2.1. The effect of CI on fatigue and listening-related fatigue

Two out of the five included studies examined fatigue and listening-related fatigue using a qualitative study design through focus group discussions (19, 53). McRackan et al. (53) developed a theoretical framework to understand fatigue in CI users who reported increased levels of general fatigue. The participants were stratified into three focus groups based on their communication abilities with their implants. The same levels of fatigue were mentioned regardless of the performance, although the increased levels of fatigue were mentioned by the middle- and high-performing groups only for complex listening situations, while the low-performance group mentioned fatigue in all communication situations (53). Davis et al. (19) used a focus group to investigate listening-related fatigue in hearing aid users and CI users to develop a theoretical framework. Listening-related fatigue might be driven by situational determinants, characteristics of the listening situation and motivation, and coping strategies used in these situations (19). Only 3 out of the 43 participants in this study were CI users, which might have limited their input. Although qualitative research is useful for understanding the concept of fatigue and listening-related fatigue, the results might not be generalizable to the CI population as only a small study population was included.

#### 3.2.2. Fatigue and listening-related fatigue in CI users compared with controls with normal hearing, hearing impaired controls and among different CI groups

##### 3.2.2.1. Scales and questionnaires

Dwyer et al. (41) investigated fatigue in a group of students consisting of CI users, hearing aid users, and controls with normal hearing with the fatigue subscale of the POMS. Additionally, Dwyer et al. (41) developed a three-item questionnaire to investigate fatigue and listening-related fatigue. They found no difference in levels of fatigue between CI users and controls with normal hearing based on the POMS but observed higher listening-related fatigue when executing the three-item Likert scale (41). The association could be confirmed by Alhanbali et al. (18) who found increased levels of fatigue on their FAS among CI users, hearing aid users, and SSD patients compared to the participants with normal hearing without difference in fatigue between the hearing impaired groups. Furthermore, FAS scores above percentile 95 of the control group were defined as extreme levels of fatigue by Alhanbali et al. (51), who found extreme levels of fatigue in 10% of the CI users. However, Dwyer et al. (41) stated that none of the CI users reported severe levels of fatigue when defined as a score that exceeds the normative means by ±1.5 SDs (41). Both studies differ in sample size, age of participants, and definition of extreme/severe fatigue. The participants in the study of Dwyer et al. (41) were all students, while Alhanbali et al. (18) included older participants. As the study population of Dwyer et al. (41) is rather specific, the results may not be generalizable to the entire CI population. The aim of the POMS is to measure different moods, with fatigue being only one of them. As a result, the POMS may not be sensitive enough to measure listening-related fatigue as a significant difference between the CI users and the participants with normal hearing could be found in the same study population when using the self-developed three-item questionnaire to examine listening-related fatigue.

The impact on fatigue after the sequential implantation of a second CI was investigated by Härkönen et al. (54). With the second CI, fatigue after the working day was reduced (54). Caution is necessary to interpret these data as only one question was used to evaluate fatigue before and after implantation.

##### 3.2.2.2. Objective measurements

All results in this section were gained using subjective measurements. There are, to the best of our knowledge, currently no studies that investigated fatigue and listening-related fatigue among CI users using objective measurements. However, it may be possible to investigate this in this population when monitoring task performance to observe the fatigue-related decrements in cognitive processing or with the use of biological markers and (electro)physiological techniques (15).

### 3.3. Association between listening effort and fatigue

Three studies investigated the association between listening effort, fatigue, and listening-related fatigue among CI users. High levels of listening effort might be associated with increased levels of fatigue and listening-related fatigue (19, 41, 51). As it was already suggested that the POMS was not sensitive enough to measure listening-related fatigue, this may explain the lack of association found between listening effort and fatigue using this questionnaire (41).

## 4. Discussion

This scoping review aimed to provide an overview of the current knowledge of listening effort, fatigue, and listening-related fatigue among CI users and the different measurement methods. Due to the heterogeneity of the methodologies used to measure this, inconclusive results were found.

The results of this scoping review show that cochlear implantation may reduce listening effort in hearing-impaired individuals. However, when listening effort in CI users was compared with controls with normal hearing, hearing impaired controls without CI, and between unilateral, bilateral, and bimodal CI users, the results of the included studies were inconclusive. A possible explanation for these inconclusive results is the variance in the study population. There are great variances in the number and age range of participants and the composition of the control group. Additionally, the CI users in the studies differ in type and brand of CI and in the distribution of unilateral, bilateral, and bimodal CI.

A second possible explanation for the inconclusive results is the use of different measurement methods. Although questionnaires, dual-task paradigms, pupillometry, and EEG measurements have good reliability, the lack of correlation between these measurements may support the idea that listening effort is a multidimensional construct, which makes it difficult to measure with only one measurement method (51). This hypothesis is supported by the Framework for Understanding Effortful Listening (FUEL) model by Pichora-Fuller et al. (7). This model stated that a mismatch of the cognitive demands and cognitive resources, allocated by multiple dimensions (e.g., arousal level, evaluation of task demands, and capacity), will lead to effort (7).

Because listening effort is a complex multidimensional construct, it is not yet clear how it should be measured. This might explain the variance in measurement methods used in the included studies and stresses the need for clarification on which method should be used to measure listening effort among CI users to support further research into these topics. This knowledge is crucial to determine the impact of hearing loss, CI, and CI parameters on listening effort. The subjective, behavioral, and physiological measurements have their advantages and disadvantages. First of all, subjective measurement techniques are quick, easy, and fast to administer in clinical practice but participants may give socially desirable answers or the answers may be influenced by their interpretation (10, 51). In the included studies, a great variance of scales and (parts of) questionnaires were used. However, comparable results were achieved when comparing CI users with controls with normal hearing when utilizing different scales and questionnaires. When investigating the difference between CI users and hearing aid users and between different CI groups, the self-reported questionnaires yielded conflicting results. These inconsistent results were also reported in the systematic review conducted by Ohlenforst et al. (24). This review aimed to investigate if hearing loss and hearing aids have an impact on listening effort (24). The authors noted a great variety in scales and questionnaires and recommended avoiding such variety to facilitate further research (24). To our knowledge, only one validated instrument has been developed specifically to measure listening effort. The Effort Assessment scale (EAS) developed by Alhanbali et al. (18) could be a potential questionnaire that is appropriate for assessing subjective listening effort among the hearing-impaired population. The EAS may be useful in clinical practice as it comprises only six questions, three from the listening effort subscale of the SSQ and three additional questions. Another strength of the EAS is that it is not specifically designed to measure listening effort in CI users, making it suitable for assessing hearing aid users and controls with normal hearing. This ensures that all research on this topic can be conducted using the same questionnaire, making it easier to compare results. Based on our review, it appears that the subscale of the SSQ as well as the EAS can measure differences in listening effort between CI users and controls with normal hearing. However, these measures do not appear to be sensitive enough to compare listening effort between CI users and hearing-impaired controls without CI or among CI groups. Another questionnaire deserving attention is the Listening Effort Questionnaire – Cochlear Implant (LEQ-CI). This questionnaire is currently in development and has not yet been validated (55). The LEQ-CI comprises 29 questions divided into four scales: effort of attending, effort of processing, effort associated with adapting and compensating for a hearing loss, and motivation. Due to the comprehensive survey and questioning of various aspects of listening effort, this questionnaire also holds promise for future research in the CI population. One disadvantage of the LEQ-CI is that it is specifically designed to measure listening effort in the CI population and therefore may be less suitable for investigating the hearing aid population and controls with normal hearing. The disadvantages of the self-reported subjective measurement methods can be avoided by using objective measurements. During a dual-task paradigm, two tasks have to be executed at the same time, resulting in this method mimicking a real-life situation where multitasking is necessary (51). This behavioral measurement is also easy to administer and interpret but may be too time-consuming to perform in a clinical setting (51). In our review, the dual-task paradigms did not reveal any differences between CI users and controls with normal hearing, hearing aid users, or between CI groups, and were not associated with subjective measures. The systematic review conducted by Ohlenforst et al. (24) revealed varying outcomes among the studies that utilized dual-task paradigms in patients with hearing loss and hearing aids. Both our study and the review by Ohlenforst et al. (24) suggest that differences in tasks may account for the differences in listening effort found. Furthermore, it remains unclear whether the absence of differences in listening effort measured with dual-task paradigms suggests that this measurement is not sensitive enough to measure small changes or differences in listening effort, or that the tasks of the paradigms assess different cognitive processing stages (10, 24). Pupillometry and EEG measurements are useful to obtain a temporal indication of the mental processing without conscious control of the participant (14, 51). All the studies included in our review reported a difference in listening effort between CI users and controls with normal hearing. These findings are consistent with the systematic review conducted by Ohlenforst et al. (24) as most of their included studies also found elevated levels of listening effort caused by hearing loss compared to controls with normal hearing and a positive effect of hearing aid amplification. As a result, it can be postulated that pupillometry and EEG measurements are useful for evaluating listening effort. However, objective measurements are more complex to execute in the clinical practice and are susceptible to external factors such as motivation, mental state, and task engagement of the CI user (14, 51). This is an important disadvantage of these measurement methods. Additionally, there are currently no standards for the objective measurements, limiting their use to within-person comparisons. Nevertheless, further exploration of the EEG measurements may prove beneficial for research purposes in order to broaden our understanding of the brain regions that are responsible for an increasement in listening effort.

It was not in our scope to discuss possible influencing factors of listening effort though inconclusive results were reported in the literature. It is currently not clear what the impact is of patient-related factors such as age, duration of hearing impairment prior to implantation, cognition, and employability on listening effort (10, 32, 42), and whether speech recognition is associated with listening effort (9–12, 32, 39). CI users with a lower working memory capacity seem to exert higher levels of listening effort (39) although Abdel-Latif and Meister (10) and Perreau et al. (42) found no association between listening effort and working memory capacity or processing speed in CI users. Previous studies could not establish whether specific CI algorithms and microphone technologies reduce listening effort or if listening effort is associated with spectral resolution (44, 56, 57). On the other hand, it has already been suggested that a slower speaking rate and the availability of a relevant semantic context may reduce listening effort in CI users (58, 59). Furthermore, listening effort seems to decrease with increasing SNR levels (12, 42, 60) although, even in favorable listening conditions, listening is still an effort for CI users (60). Considering that listening remains an effort for CI users and the higher listening effort reported in CI compared to controls with normal hearing, it is interesting to further explore the influencing factors as there is still much ambiguity about it.

Only five studies investigated the effect of fatigue or listening-related fatigue in CI users, which highlights the need for further research as no conclusions can be made based on the available literature. In addition, studies also differ in study population, methodology, and measurement methods. Furthermore, there is a need for standardized terminology regarding fatigue and listening-related fatigue as well as a standardized methodology to measure it. Fatigue is a complex phenomenon that encompasses both short-term and long-term fatigue and can be influenced not only by hearing loss but also by factors such as personal motivation, emotions, lifestyle, and coping strategies (61–64). Similarly to the measurement methods for evaluating listening effort, subjective and objective measurements can be used to determine fatigue. In the studies included in our review, both the FAS and the subscale of the POMS were employed to measure fatigue, however, contradictory outcomes were observed (18, 41). The study conducted by Hornsby et al. (15) in hearing-impaired patients supported the lack of difference found by Dwyer et al. (41) between hearing-impaired individuals and controls with normal hearing, as no difference was found when administering the POMS and the multi-dimensional fatigue symptoms inventory-short form, which is a fatigue questionnaire developed to investigate cancer-related fatigue (65). Recently, a promising validated listening-related fatigue questionnaire, the VFS-A, was developed by Hornsby et al. (20). The VFS-A can be useful to execute along with objective measurements such as monitoring the task performance, biological markers, and (elektro)physiological measurements. Studies that conducted objective measurements to investigate fatigue and listening-related fatigue among the hearing-impaired population are scarce (51, 66). To the best of our knowledge, only one study implemented a dual-task paradigm to monitor task performance and proposed a decrease in fatigue when utilizing hearing aids (67). This measurement method has some limitations as boredom or a lack of engagement can also lead to a decrease in task performance, or the learning effect can lead to an absence of a decrease in task performance while fatigue still develops (15, 51, 61). In the review conducted by Holman et al. (66), which investigated the effect of hearing loss on listening-related fatigue, all studies except one used subjective measures. Most of the studies included in the review reported elevated levels of fatigue or listening-related fatigue in the hearing-impaired population (66). Nevertheless, it has been suggested that decreased cortisol levels are seen when fatigue occurs (68–70). While Dwyer et al. (41) measured the cortisol levels, they did not detect any difference between CI users and controls with normal hearing. This finding is supported by Hick and Tharpe (71) and Kramer et al. (72), suggesting that measuring cortisol levels might not be sensitive enough to detect differences in fatigue. In contrast, Bess et al. (73) discovered elevated cortisol levels among children with hearing impairments compared to peers with normal hearing. Other objective measurements used to investigate fatigue are ERP measurements, in which Key et al. (74) identified a difference in the P300 before and after performing a speech task. Similar to these studies, our review did not reveal a consensus regarding the measurement methods of fatigue. The absence of correlation between the measurement methods may indicate a low reliability and sensitivity of these measurements or that fatigue, similar to listening effort, is a multidimensional construct, possibly consisting of different types of fatigue (e.g., physical fatigue, mental fatigue, emotional fatigue, and vigor) (65). One important limitation requiring further research attention is the absence of objective measurements to assess listening-related fatigue, as they are currently unavailable, to the best of our knowledge. An important limitation of a scoping review is the lack of exclusion of studies based on their methodology, which may limit this review by the quality of the included studies. Moreover, a language bias could occur as possible interesting studies in languages other than Dutch or English were excluded.

## 5. Conclusion

Recently, there has been an increased interest in listening effort, fatigue, and listening-related fatigue to provide proper rehabilitation. Research in these areas is in its early stages. Current research employs a wide range of measurement methods, which makes it difficult to compare studies. The utilization of a uniform definition and measurement of listening effort, fatigue, and listening-related fatigue will enable further research to be compared. However, a standardized measurement method has not yet been developed for CI users or hearing-impaired groups. With the rising number of CI users, there is a need to review the current literature that focuses on this population. This scoping review suggests that CI users experience higher levels of listening effort, fatigue, and listening-related fatigue. When it is clear how listening effort and fatigue should be measured, further research can determine the impact of a CI on listening effort, fatigue, and listening-related fatigue as well as the factors that contribute to a decrease or increase in these effects. Furthermore, there is a need for guidelines to measure listening effort, fatigue, and listening-related fatigue in clinical practice as this might eventually optimize the current rehabilitation of the CI population.

## Author contributions

CP: Data curation, Formal analysis, Investigation, Visualization, Writing – original draft, Writing – review & editing. LJ: Conceptualization, Supervision, Writing – review & editing. ML: Conceptualization, Supervision, Writing – review & editing. GM: Writing – review & editing. AG: Writing – review & editing. OV: Writing – review & editing. VR: Conceptualization, Supervision, Writing – review & editing.

## Appendix 1

Summary of the study characteristics classified in alphabetic order.

**Table:**

	Participants				Measurement methods	Outcome
Author (year of publication)	Total	CI users (age range, males, females)	CI	Controls (age range, males, females)		
**LISTENING EFFORT**						
Abdel-Latif and Meister (10)	28	14 (39–83, 6, 8)	3 unilateral	14 NH (−, 3, 11)	Adaptive Categorical Listening Effort Scaling Dual-task paradigm	Subjective measurement: higher LE in CI users compared to NH participants Objective measurement: no difference in LE between CI users and NH participants No correlation between subjective and objective measurement
			11 bilateral			
Alhanbali et al. (18)	200	50 (55–80, 26, 24)	50 unilateral	50 HA (55–85, 31, 19)	Effort Assessment scale	Higher LE in hearing-impaired individuals compared to NH participants No difference in LE between the hearing-impaired groups Significant correlation between LE and fatigue
				50 SSD (58–80, 30, 20)		
				50 NH (55–78, 22, 28)		
Burg et al. (49)	12	12 (25–78, −, −)	12 bilateral	-	Pupillometry	Higher LE in bilateral condition compared to unilateral condition
Devocht et al. (46)	15	15 (−, 8, 7)	15 bimodal	-	13-point scale	Further reduction of LE for bimodal listening situation compared to a CI alone listening situation
Dillon et al. (3)	20	20 (23–66, −, −)	20 unilateral SSD	-	Speech, Spatial, and Qualities of Hearing Scale	LE decreases after cochlear implantation in SSD patients
Dimitrijevic et al. (36)	10	10 (23–74, 4, 6)	2 unilateral	-	NASA Task Load Index Electroencephalogram	Positive association between LE and left frontal IFG alpha power Negative association between LE and speech envelope auditory cortex coherence in the 2–5 Hz range
			8 bilateral			
Dingemanse and Goedegebure (39)	103	48 (29–89, 31, 17)	48 unilateral	55 NH (30–77, 22, 33)	Speech, Spatial, and Qualities of Hearing Scale	Higher LE in CI users compared to NH participants
Dwyer et al. (40)	87	20 (32.8–75.2, −, −)	20 unilateral	21 NH (26.6–73.3, −, −)	Speech, Spatial, and Qualities of Hearing Scale	Higher LE in hearing-impaired individuals compared to NH participants No difference in LE between the hearing-impaired groups LE was significantly lower in the bilateral CI and bimodal group compared to the UHL group without CI
				30 SSD (25.3–75.9, −, −)		
				16 HA (60.3–77.4, −, −)		
Dwyer et al. (41)	14	6 (23–32, 1, 5)	1 unilateral	8 NH (1, 7, 21–30)	4-point Likert scale	Higher LE in hearing-impaired individuals compared to NH participants Correlation between subjective LE and listening-related fatigue
			4 bilateral	2 HA (23–23, 0, 2)		
			1 bimodal			
Farinetti et al. (8)	116	116 (−, 47, 69)	54 unilateral	-	Speech, Spatial, and Qualities of Hearing Scale	No difference in LE for bimodal listeners compared to unilateral CI users
			62 bimodal			
Finke et al. (11)	26	13 (43–75, 6, 7)	-	13 NH (44–74, −, −)	5-point Likert scale Electroencephalogram	Higher subjective and objective LE in CI users compared to NH participants
Gifford et al. (45)	11	11 (40–75, 7, 4)	9 unilateral	-	Visual analog scale	Lower LE for bilateral EAS participants compared to bimodal participants
			2 bilateral			
Hughes et al. (38)	17	11 (42–84, −, −)		4 HA (53–84, −, −)	Focus group discussion	Development of a theory of LE in adults with severe-to-profound hearing loss with a CI and CI candidates
				2 significant others (60–74, 0, 2)		
Lopez et al. (47)	40	40 (23–79, 17, 23)	40 unilateral	-	Speech, Spatial, and Qualities of Hearing Scale	LE decreases after cochlear implantation without a difference between SSD and asymmetric hearing loss group
McRackan et al. (32)	371	371 (18–89, 149, 222)	87 unilateral	-	Cochlear Implant-Related Quality of Life	LE is positively associated with bilateral implantation
			96 bilateral			
			188 bimodal			
Noble et al. (9)	145	145 (−, −, −)	70 unilateral	-	Speech, Spatial, and Qualities of Hearing Scale	Lower LE ratings in participants with a bilateral CI compared to participants with a unilateral CI or bimodal CI
			36 bilateral			
			39 bimodal			
Paul et al. (12)	16	16 (23–75, 7, 9)	6 unilateral	-	10-point scale Electroencephalogram	Inverted U-shaped relation between LE and alpha power in parietal sensors No relationship between LE and alpha oscillations in the left IFG
			3 bilateral			
			7 bimodal			
Perreau et al. (42)	46	34 (21–77, 14, 20)	22 unilateral	12 NH (1, 11, 42, 46–60)	Speech, Spatial and Qualities of Hearing Scale Dual-task paradigm	Subjective measurement: higher LE in CI users compared to NH participants, no difference in LE between unilateral or bilateral CI users Objective measurement: no difference in LE between the CI users and normal hearing participants, no difference in LE between unilateral and bilateral CI users No correlation between subjective and objective measures
			12 bilateral			
Sladen et al. (44)	16	16 (29–77, 6, 11)	11 bilateral	-	Ease of Listening Scale Dual-task paradigm	Subjective measurement: no difference in LE between bilateral and bimodal CI users Objective measurement: no difference in LE between bilateral and bimodal CI users
			5 bimodal			
Wagner et al. (14)	29	15 (30–73, 9, 6)	15 unilateral	14 NH (5, 9, 24–70)	Pupillometry	Smaller phasic and slower decrease in ERPD for CI users compared to normal hearing participants Function differs in time course, shape, and location of peaks between CI and NH participants Variation between different CI users in response magnitude, time course, morphology, latencies, and number of peak dilations
Yüksel et al. (48)	20	20 (16–25, 7, 13)	12 unilateral	-	Dual task-paradigm	No difference in listening effort between unilateral and bimodal CI users
			8 bimodal			
**FATIGUE**						
Alhanbali et al. (18)	200	50 (55–80, 26, 24)	50 unilateral	50 HA (55–85, 31, 19)	Fatigue Assessment Scale	Increased fatigue levels in hearing-impaired individuals compared to NH participants No difference in fatigue between the hearing-impaired individuals Significant correlation between LE and fatigue
				50 SSD (58–80, 30, 20)		
				50 NH (55–78, 22, 28)		
Davis et al. (19)	43	3 CI (25–70, −, −)	1 bilateral	40 HA (20–77, −, −)	Focus group discussion	Development of a theoretical framework for listing-related fatigue Association between LE and listening-related fatigue
			2 bimodal			
Dwyer et al. (41)	14	6 (23–32, 1, 5)	1 unilateral	8 NH (1, 7, 21–30)	Profile of Mood States 5-point Likert scale Salivary Cortisol level	No significant difference in general fatigue between hearing impaired and NH participants More listening related-fatigue in hearing-impaired participants compared to NH participants Correlation between subjective LE and listening-related fatigue No association between subjective LE and fatigue
			4 bilateral	2 HA (23–23, 0, 2)		
			1 bimodal			
Härkönen et al. (54)	15	15 (19–58, 6, 9)	15 bilateral	-	Undefined	Sequential implantation of the second CI reduces fatigue after a workday
McRackan et al. (53)	23	23 (46.2–84.2, 13, 10)	3 unilateral	-	Focus group discussion	Low-performance CI users reported that they often felt fatigue after a full day of listing while, the middle- and high-performing CI users reported the same amount of fatigue but in complex listening environments
			14 bilateral			
			6 bimodal			
			2 EAS			

CI, cochlear implant; NH, normal hearing; LE, listening effort; HA, hearing aid; SSD, single sided deafness; EAS, Electric-Acoustic Stimulation system; IFG, inferior frontal gyrus; ERPD, event-related pupil dilation.
